# Comparative analysis of genes frequently regulated by drugs based on connectivity map transcriptome data

**DOI:** 10.1371/journal.pone.0179037

**Published:** 2017-06-02

**Authors:** Xinhua Liu, Pan Zeng, Qinghua Cui, Yuan Zhou

**Affiliations:** 1 Department of Biochemistry and Molecular Biology, School of Basic Medical Sciences, Tianjin Medical University, Tianjin, China; 2 Department of Biomedical Informatics, School of Basic Medical Sciences, Peking University, Haidian District, Beijing, China; 3 Centre for Noncoding RNA Medicine, MOE Key Lab of Cardiovascular Sciences, School of Basic Medical Sciences, Peking University, Haidian District, Beijing, China; Tianjin University, CHINA

## Abstract

Gene expression is perturbated by drugs to different extent. Analyzing genes whose expression is frequently regulated by drugs would be useful for the screening of candidate therapeutic targets and genes implicated in side effect. Here, we obtained the differential expression number (DEN) for genes profiled in Affymetrix microarrays from the Connectivity Map project, and conducted systemic comparative computational analysis between high DEN genes and other genes. Results indicated that genes with higher down-/up-regulation number (down_h/up_h) tended to be clustered in genome, and have lower homologous gene number, higher SNP density and more disease-related SNP. Down_h and up_h were significantly enriched in cancer related pathways, while genes with lower down-/up-regulation number (down_l/up_l) were mainly involved in the development of nervous system diseases. Besides, up_h had lower interaction network degree, later developmental stage to express, higher tissue expression specificity than up_l, while down_h showed reversed tendency in comparison with down_l. Together, our analysis suggests that genes frequently regulated by drugs are more likely to be associated with disease-related functions, but the extensive activation of conserved and widely expressed genes by drugs is disfavored.

## Introduction

Identification of genes competent to be drug targets is one initial step for drug discovery [1]. For example, *TP53*, also known as *p53*, is one of the most well known tumor suppressor genes in most of cancers [2–5], and some relevant drugs have also arisen [6–8]. Mutations of *PIK3CA* were found to be closely associated with the development of glioblastomas, gastric cancer, breast cancer and lung cancer, which could be an important therapeutic target for them [9]. In the study of Spires *et al*, gene-environment interactions were considered to play important roles in neurodegenerative disease like Alzheimer’s disease and Huntington’s disease [10].

Compared with studies about genetic associations with diseases, such as genome-wide association study (GWAS), gene expression profiles were more accessible and easier to obtain and analyze. As indicated by the statistics of Gene Expression Omnibus (GEO, https://www.ncbi.nlm.nih.gov/geo/) database, rapid advent of high-throughput gene expression quantification techniques has brought about the large amount of accumulation of transcriptome data. These transcriptome data are valuable resource for both pathophysiology study and drug discovery. For example, Shin *et al* developed GENT to compare expression status of genes between normal and tumor tissues profiled by Affymetrix U133A or U133plus2 microarray platforms [11]. GOBO is a database for breast cancer in which multiple analyses on transcriptome data, including survival analysis, comparative analysis and co-expression analysis could be performed [12]. What’s more, there emerged some drug target gene databases according to the drug-induced gene expression changes, e.g. DSigDB (http://tanlab.ucdenver.edu/DSigDB/DSigDBv1.0/) [13] and DGIdb (http://dgidb.org/) [14]. Finally, the integration of gene expression data significantly contributed to the development of accurate drug target prediction tool [15]. For example, Kutalik *et al* developed a modular approach for the integration of large-scale gene expression and drug-response data to predict drug-target interactions [16]. All above studies would promote our knowledge about specific roles of genes in diseases and more importantly potential target genes of a specific drug.

However, the gene expression is not uniformly perturbated by drugs, and it is likely that expression of some genes is widely responsive to various drugs. Analyzing genes whose expression is frequently regulated by drugs would be useful for the screening of candidate therapeutic targets and genes implicated in side effect. However, without sizable and well-controlled transcriptome dataset which covers multiple drug treatment condition, such analysis was not feasible. Recently, the Connectivity Map (CMAP) project [17, 18] has accumulated thousands of whole transcriptome expression profiles detected through Affymetrix Human Genome U133 Array with controlled protocol. This transcriptome dataset represents several human cell lines (mainly MCF7, ssMCF7, PC3, HL60 and SKMEL5) and treatments with 1,309 bioactive small molecules. Through CMAP, we could infer the functional connections between drugs, genes and diseases. In this study, we first identified genes with higher and lower differential expression number (DEN), i.e. genes more or less likely differentially expressed among various treatment condition. Systematic comparative analysis for these two types of genes has been performed. The differences in evolution, functions, baseline expression, and interaction network topology have been analyzed and summarized, which would provide helpful resource for prioritizing the favorable drug targets.

## Materials and methods

### Identification of genes with higher and lower DEN

**Fig 1:**
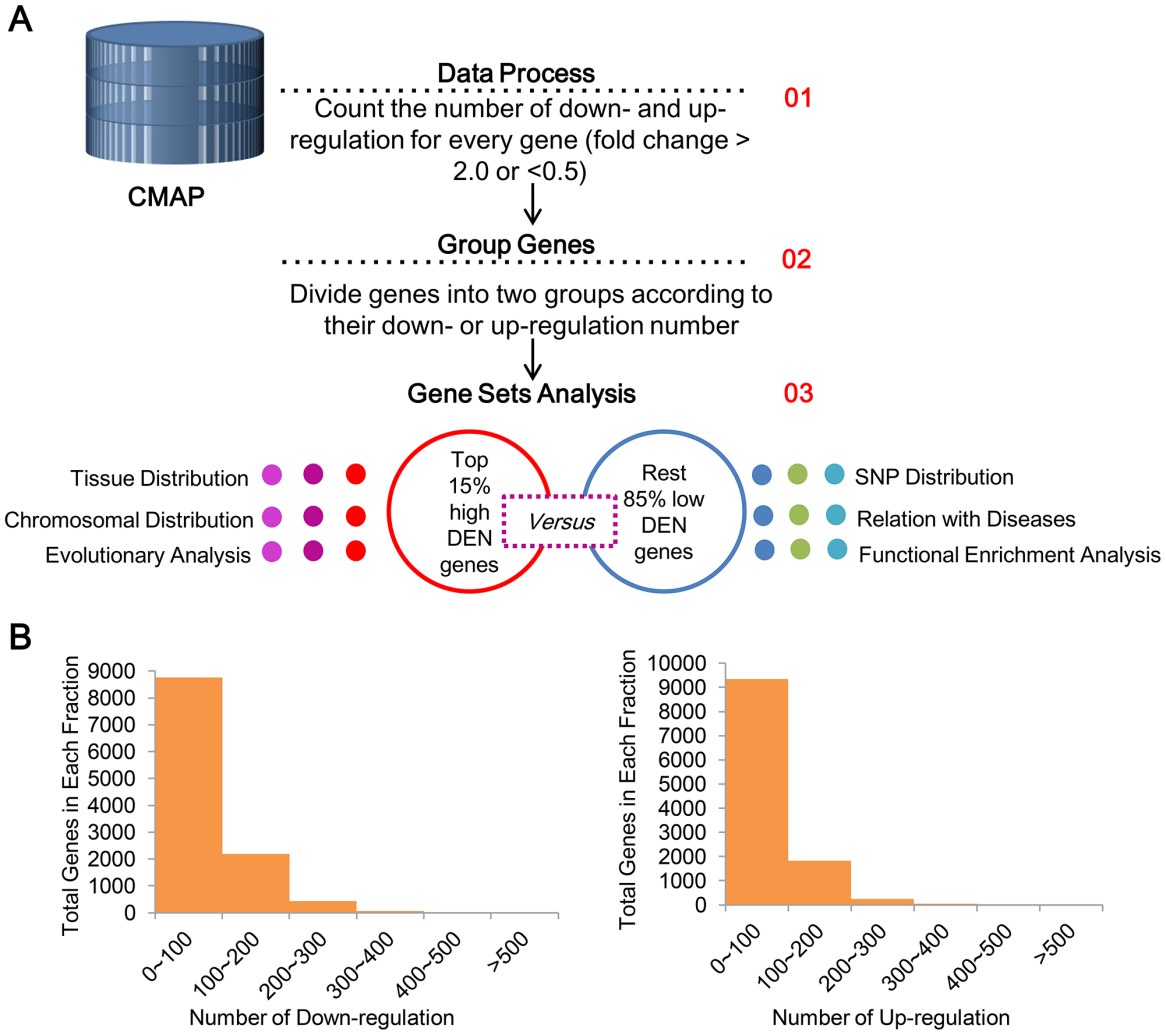
The overall view of the analysis. (A) The pipeline for the calculation of DEN of every gene from the CMAP dataset and the following computational analysis. (B) The distribution of down-regulation number (left) and up-regulation number (right) among the analyzed genes.

We downloaded the fold change matrix from the Connectivity map (CMAP) database (http://portals.broadinstitute.org/cmap), in which rows and columns represent probesets and treated cell line samples respectively, and each cell in the matrix is the logarithmic transformed fold change (lnFC) of the probe in the corresponding samples (in comparison with its matched control cell line). In this study, lnFC > 0.69 or <-0.69 cutoffs (which were equivalent to fold change > 2 or < 0.5 cutoffs) were used for the determination of significantly up- and down-regulation in each treated cell line sample. Then the numbers of significantly up- and down-regulated samples for each probe were counted as the DENs. We mapped the probes to genes and divided genes into four groups according to their DENs. More specifically, within each gene set used by the following analysis, the genes with top 15% highest up-regulation numbers were denoted as up_h set, while the rest 85% genes were denoted as up_l set. The down_h and down_l sets were defined in similar fashion. The top 15% cutoff was deduced according to the observed DEN distributions (Fig 1).

### Analysis of chromosomal distribution and chromosomal distance of genes

We counted the down- and up-regulation number of genes in every chromosomal for the analysis of chromosomal distribution of DEN. We calculated the chromosomal distance between gene pairs from the same chromosome based on their genome coordinates. The statistical significance between up_h and up_l, and that between down_h and down_l were analyzed by Wilcoxon test.

### Tissue-specific expression and earliest expression stage analysis

To explore the differences in tissue-specific expression pattern between genes with higher and lower DEN, we calculated the tissue expression specificity (TES) scores for up_h, up_l, down_h and down_l respectively based on Su's [19] dataset (GEO accession number: GDS590). For each gene, TES was represented by the ratio of the largest and sum expression value of the 79 human tissues in Su's datasets. We also obtained the earliest expression (developmental) stage for every gene from OGEE database (http://ogee.medgenius.info/browse/) [20], and calculated the fraction of genes at every stage. The statistical significance for TES and earliest expression stage comparisons was determined by Wilcoxon test and Chi-squared test, respectively.

### Evolution and functional enrichment analysis

Homologous gene number and phyletic age from Homologene database [21] and OGEE database [20] were adopted to represent their evolution characteristics. And the statistical significance was determined by Wilcoxon test and Chi-squared test, respectively.

To investigate functional difference between high DEN genes and low DEN genes, we conducted functional enrichment analysis for each of four gene sets by DAVID tool (https://david.ncifcrf.gov/) [22]. Biological process terms and KEGG pathways satisfied the criteria of *P-Value* < 0.05 and the minimum hits > 2 were considered to be significant enriched terms.

### SNP densities, disease-related SNPs of genes and disease genes

We downloaded the genome coordinates for all of the single nucleotide polymorphisms (SNPs) and protein-coding genes from the Ensembl database (http://www.ensembl.org) [23] and mapped SNPs to the corresponding genes. The SNP density for a specific gene was defined as the total number of SNPs mapped to this gene divided by the length of this gene. Besides, we obtained the disease-related SNPs (dSNPs) from the ClinVar (https://www.ncbi.nlm.nih.gov/clinvar/) [24] and Human Gene Mutation Database (HGMD, http://www.hgmd.cf.ac.uk/) [25] with the removal of SNPs without dbSNP ID, as well as those flagged as "protective", "(Likely) Benign", "Uncertain significance", "conflicting data from submitters", "other" and "not provided". The number of dSNPs contained in up_h, up_l, down_h and down_l were counted and the corresponding fractions of dSNPs were calculated. The statistical significances of SNP density and dSNP fraction comparison were determined by Kolmogorov-Smirnov test and Chi-squared test, respectively.

### Protein-protein interaction network degree and subcellular localization analysis

We downloaded the human protein-protein interaction (PPI) network from the BioGRID database (release 3.4.134) (https://thebiogrid.org/) [26]. Genetic interactions and covalent interactions between ubiquitin and its substrates (i.e. ubiquitination) were removed. The interaction network degree was defined as the number of interaction partners in PPI network. Differences of degree distribution between up_h and up_l, down_h and down_l were compared by Wilcoxon test. We extracted genes with subcellular localization of extracellular region, membrane, cytoplasm and nucleus from the four group of genes based on gene ontology terms. The proportion of genes in each subcellular localization was compared by Chi-square test.

## Results and discussion

### Distribution of DEN

The overall framework of this study was illustrated in Fig 1A. The fold change matrix obtained from CMAP contained 6,101 samples and 22,140 probes (which represented 12,637 unique Entrez genes). Power-law distributions were observed for both down- and up-regulation numbers as shown in Fig 1B, where most of genes have the up- or down-regulation number smaller than 100 and only a small proportion (about 15%) of genes have higher DENs. Fig A and Fig B in S1 Fig illustrated the fold changes of the top 20 and last 20 genes from the distributions of down-regulation number and up-regulation number, respectively. The down_h genes (or up_h genes) can be clearly distinguished from the down_l genes (or up_l genes) in these heatmaps. It is also noteworthy that there is no definite distinction between the up_h and down_h genes. Several up_h genes in the heatmap (e.g. *SUGP1*, *ENFA3*, *TUBA3C* and *ZNF354A*) are also down_h genes, and *vice versa*. Indeed, about one third of the up_h and down_h genes are shared (Fig C in S1 Fig), indicating some genes are prominently responsive to a variety of drug treatments and could act differentially in response to different treatment conditions.

### Correlation between chromosomal distribution, chromosomal distance and DEN

**Fig 2:**
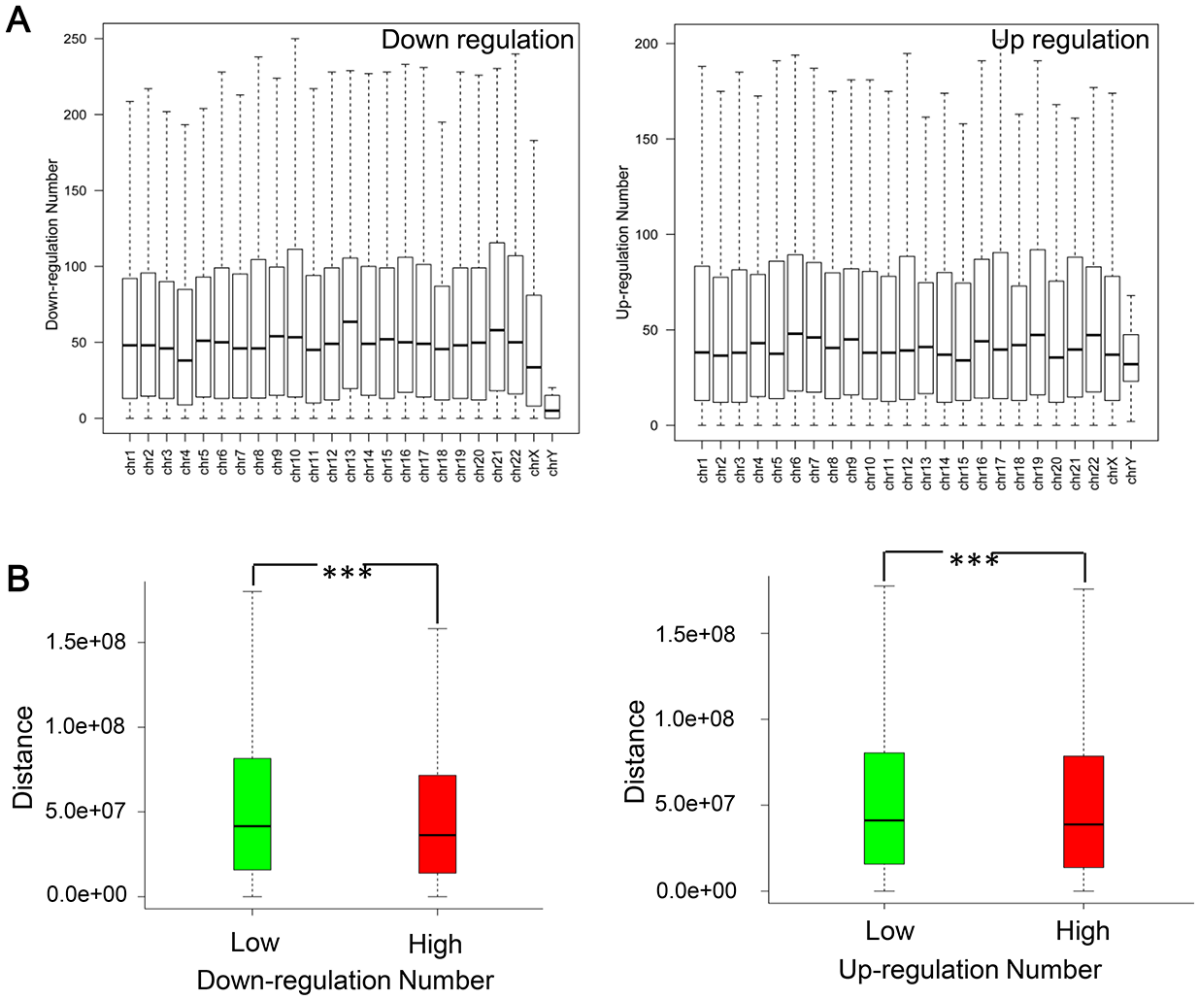
The chromosomal distribution of differentially regulated genes. (A) Distribution of down-regulation (left) and up-regulation (right) numbers across different chromosomes. (B) Chromosomal distance comparison between down_l versus down_h (left) and up_l versus up_h (right). ***, *P-Value* < 0.001by Wilcoxon test.

Similar overall chromosomal distributions for down-regulation numbers and up-regulation numbers were observed (Fig 2A). Only Chromosome Y showed significantly larger up-regulation numbers than down-regulation numbers (*P-Value* = 2.931e-08, Wilcoxon test). Chromosome-wise comparison indicated that such divergence was actually reflecting the lower frequency of down-regulation of chromosome Y genes. Our previous study also showed that the expression levels of male-specific genes were significantly higher in most of tissues [27]. Therefore, it is plausible that the expression on male-specific genes should be robust in order to sustain normal physiology.

Chromosome-wise comparison also suggested no prominent divergence of up-/down-regulation numbers between chromosomes for most cases. Nevertheless, after more detailed investigations, we found that the intra-chromosomal distribution of genes with high DEN was not random. As shown in Fig 2B, chromosomal distances among down_h gene pairs and up_h gene pairs from the same chromosome were significantly smaller than those among down_l gene pairs and up_l gene pairs (down_l versus down_h, *P-Value* = 3.243e-184; up_l versus up_h, *P-Value* = 2.482e-32, Wilcoxon test). The results indicated that genes which are more likely regulated by drugs tend to be clustered together on the chromosome.

### The differences in baseline expression pattern

**Fig 3:**
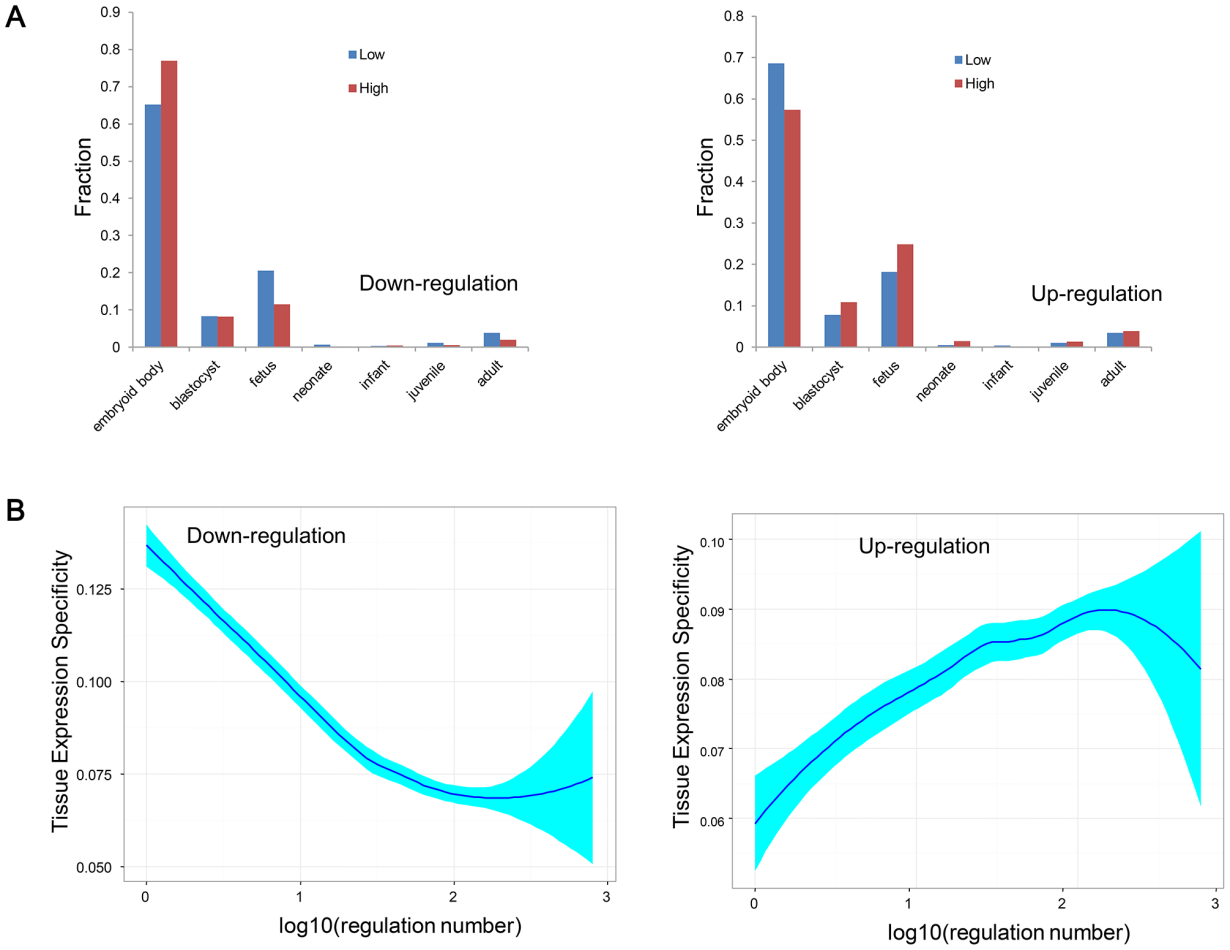
The comparison of baseline expression. (A) Comparison of earliest expression stage between down_l versus down_h (left) and up_l versus up_h (right). (B) The correlation between tissue expression specificity and up/down-regulation number. The correlation curve is plotted by using the LOESS smoothing techniques and the shade indicates the confidence interval.

We first analyzed the earliest expression stage (EES) to test whether the high DEN genes prefer to be expressed in the more specialized tissue or not (Fig 3A). Here, we divided the development process into seven stages, i.e. embryoid body, blastocyst, fetus, neonate, infant, juvenile and adult in the developmental stage order. Earlier EES indicated their more generalized functions and later EES indicated their more specialized functions. We found that the down_h tended to be expressed earlier than down_l (Chi-squared test, *P-Value* = 2.765e-14). However, we also found that the up_h tended to be expressed later than up_l (Chi-squared test, *P-Value* = 2.350e-12). We further validated such discrepancy by analyzing the tissue expression specificity (TES) scores of genes and the result recapitulated the opposite relationship between down- and up-regulation numbers with TES scores. TES scores of down_h were significantly lower than those of down_l (Wilcoxon test, *P-Value* = 2.526e-7), while the TES scores of up_h were significantly higher than those of up_l (Wilcoxon test, *P-Value* = 2.441e-34). Correlation analysis revealed the overall negative correlation between down-regulation number and TES (Fig 3B left, Spearman correlation coefficient = -0.187, *P-Value* = 4.459e-88), but overall positive correlation between up-regulation number and TES (Fig 3B right, Spearman correlation coefficient = 0.250, *P-Value* = 1.500e-158). Therefore, our analysis indicat that the down_h prefer wider gene expression but up_h tend to restrict their expression in more specialized tissues.

### Evolution and enriched functions of high DEN genes

**Fig 4:**
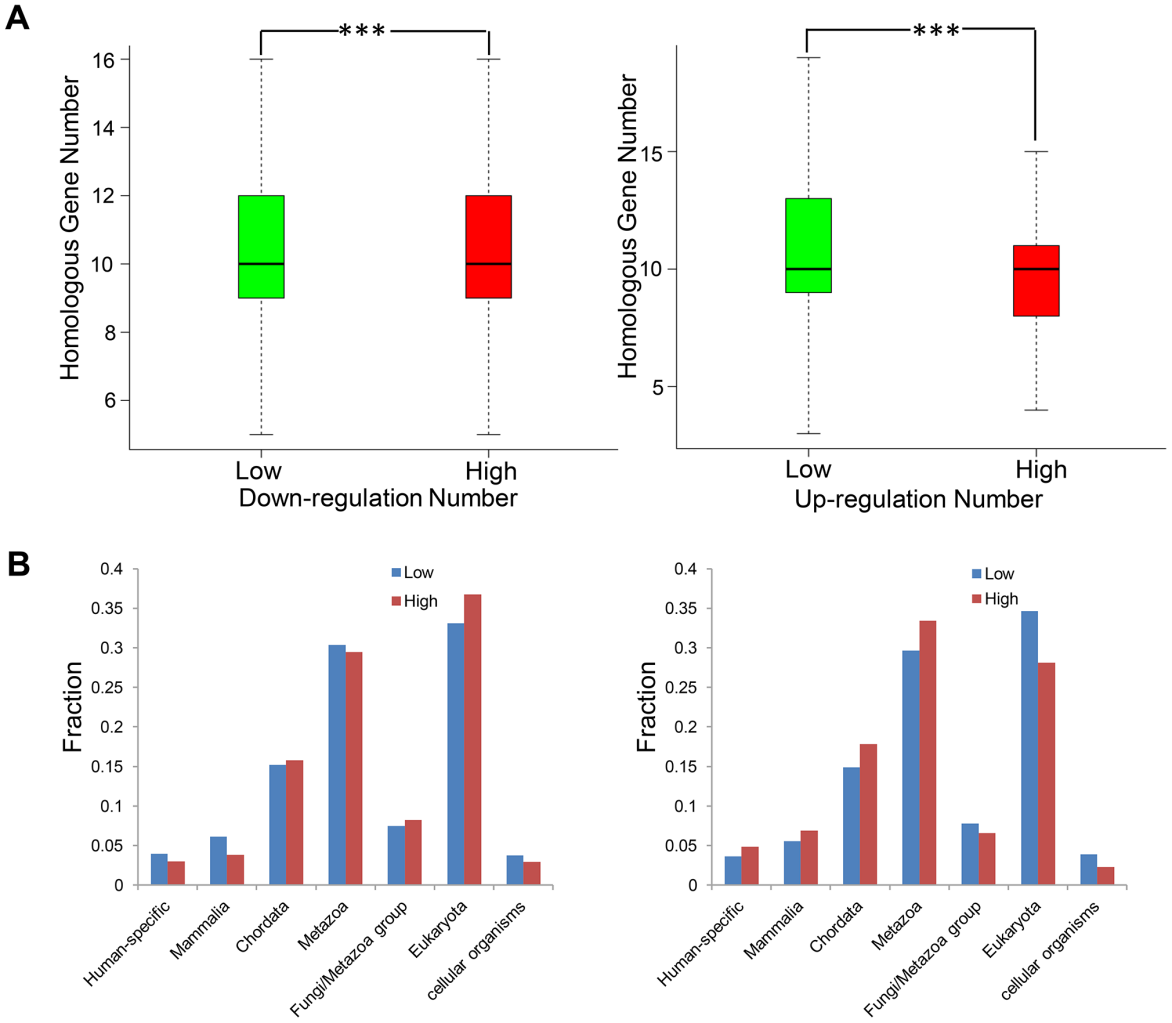
The evolutionary characteristic of genes with high DEN. (A) Comparison of homologous gene number between down_l versus down_h (left) and up_l versus up_h (right). ***, *P-Value* < 0.001 by Wilcoxon test. (B) Histogram comparing the fraction of genes in each phyletic age group.

Evolutionary characteristic and conservation are important characteristics of gene function and critical for the screening of therapeutic targets of specific disease. We first compared the homologous gene number between high DEN genes and other genes. As shown in Fig 4A, the homologous gene number of down_l and up_l were significantly higher than that of down_h and up_h (Wilcoxon test, down_l versus down_h; *P-Value* = 5.942e-5, up_l versus up_h, *P-Value* = 3.360e-24), which indicated that drug-regulated genes seemed less conservative than other genes. However the homologous gene number could be confounded by paralogs and taxonomy bias, thus we further explored the relationship between DEN and phyletic age. Interestingly, significant divergence between up_h and down_h was observed again (Fig 4B). Generally, down_h were more likely to be first presented in early eukaryotes but less likely to be mammalian- or human-specific, when compared with down_l (Chi-squared test on overall phyletic age distribution, *P-Value* = 3.760e-4). On the other hand, the up_h were clearly more enriched in mammalian- or human-specific genes than up_l (Chi-squared test on overall phyletic age distribution, *P-Value* = 1.199e-9).

We further compared the enriched functional terms between high DEN genes and other genes. It is noteworthy that simply comparing the long list of up_h, up_l, down_h and down_l would not obtain any specific term due to their large gene set sizes. Therefore, we performed functional enrichment analysis for the top (T1000D and T1000U) and last 1000 genes (L1000D and L1000U) in the DEN distribution instead. We found most of the enriched GO terms and KEGG pathways of T1000D and T1000U are closely related with the processes of cancer development, such as pathways in cancer, p53 signaling pathway, colorectal cancer, and small cell lung cancer. Whereas, processes closely associated with the development of nervous system diseases, for example nicotine addiction, Parkinson's disease, Huntington's disease, Alzheimer's disease, were found to be significantly enriched in L1000D and L1000U (S2 and S3 Figs). The significant enrichment of cancer-related processes in T1000U and T1000D might be due to the fact that the samples used for generating the expression profiles in CMAP were all cancer cell lines, including MCF7, ssMCF7, PC3, HL60 and SKMEL5. In such dataset, the cancer-related genes and pathways are more likely to be differentially expressed, thus have higher DENs. On the other hand, there were some clinical or epidemiological observations implying the anti-correlation between cancer and some nervous system diseases. For example, by surveying the data from Framingham Heart Study, Driver *et al* found that the cancer survivors were unlikely to have Alzheimer's disease, and *vice versa* [28]. The anti-correlated incidence and risk between cancer and Alzheimer's disease were also observed in another large population study [29]. Moreover, the Parkinson's disease-associated gene *PARK2* was also frequently mutated in tumors. Interestingly, *PARK2* deletion was antic-correlated with the amplification of several oncogenes like *CDK4* [30]. The detailed molecular mechanisms underlying such anti-correlation are not clear, but one plausible explanation is that when the transcriptome regulation of cancer-related pathways are activated (resulting the enrichment in high DEN genes), the transcriptome regulation of nervous system diseases-related pathways are repressed (resulting the enrichment in low DEN genes). Further studies about the mechanism for why high DEN genes and low DEN genes were mainly associated with different diseases would promote our understanding about the relationship between cancer pathways and nervous system disease pathways.

### Relationship with disease-associated SNPs

**Fig 5:**
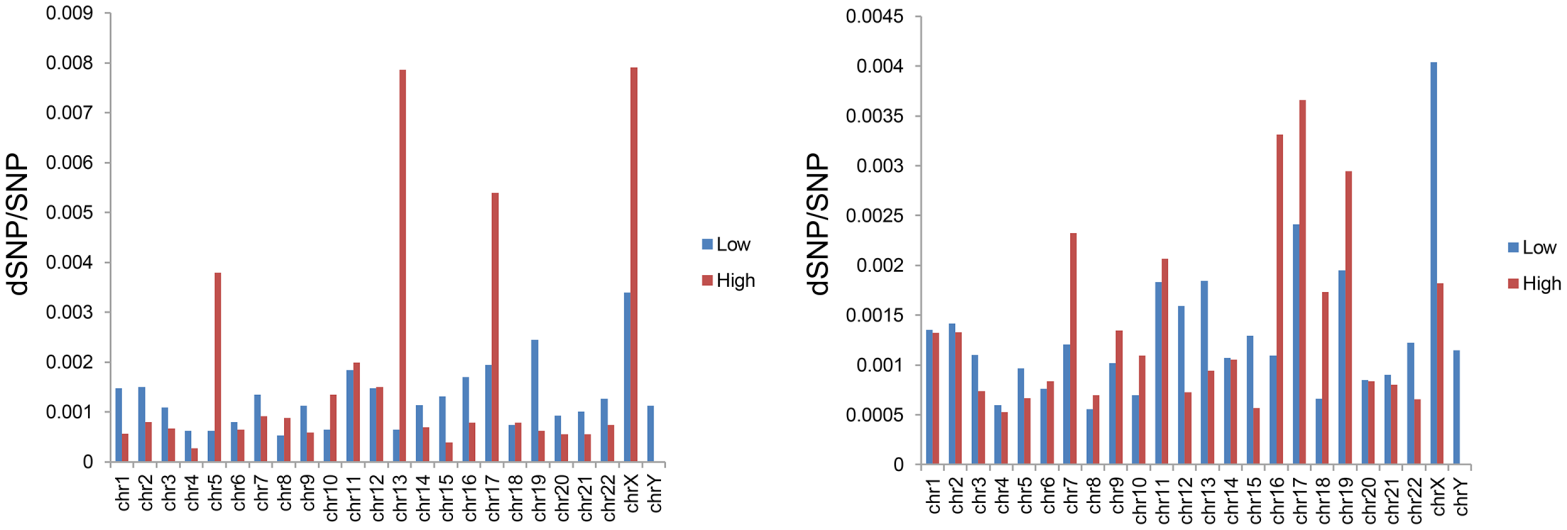
Comparison of disease-related SNPs. Proportion of dSNPs in total of SNPs of down_l versus down_h (left) and up_l versus up_h (right) were listed based on chromosomal distribution of dSNPs.

SNP is one of the most common gene mutation types and involves the progression of many diseases. The number of SNPs, particular disease-related SNPs (dSNP) contained in a gene is important indication for its functions and disease association. In this study, we first calculated the SNP density for every gene according to the method described in Materials and Methods section, and then grouped SNP density by DEN and chromosome locations. The overall SNP densities of down_l and up_l are lower than that of down_h and up_h (down_l versus down_h, *P-Value* = 0.02805; up_l versus up_h, *P-Value* = 0.00133, as indicated by Kolmogorov-Smirnov test). Nevertheless, the proportion of dSNP in SNP of down_h and up_h are significantly higher than that of down_l and up_l for most of chromosomes (Fig 5). Besides, we tested if the overall proportion of dSNP was associated with DEN through Fisher exact test, and the results indicated that the proportions of dSNP in down_h and up_h were all significantly higher than those in down_l and up_l (down_h versus down_l, *P-Value* = 0; up_h versus up_l, *P-Value* = 0). All of these results indicated that genes with higher DEN might be more likely to be associated with the progression of diseases.

### Network topology and subcellular localization analysis

**Fig 6:**
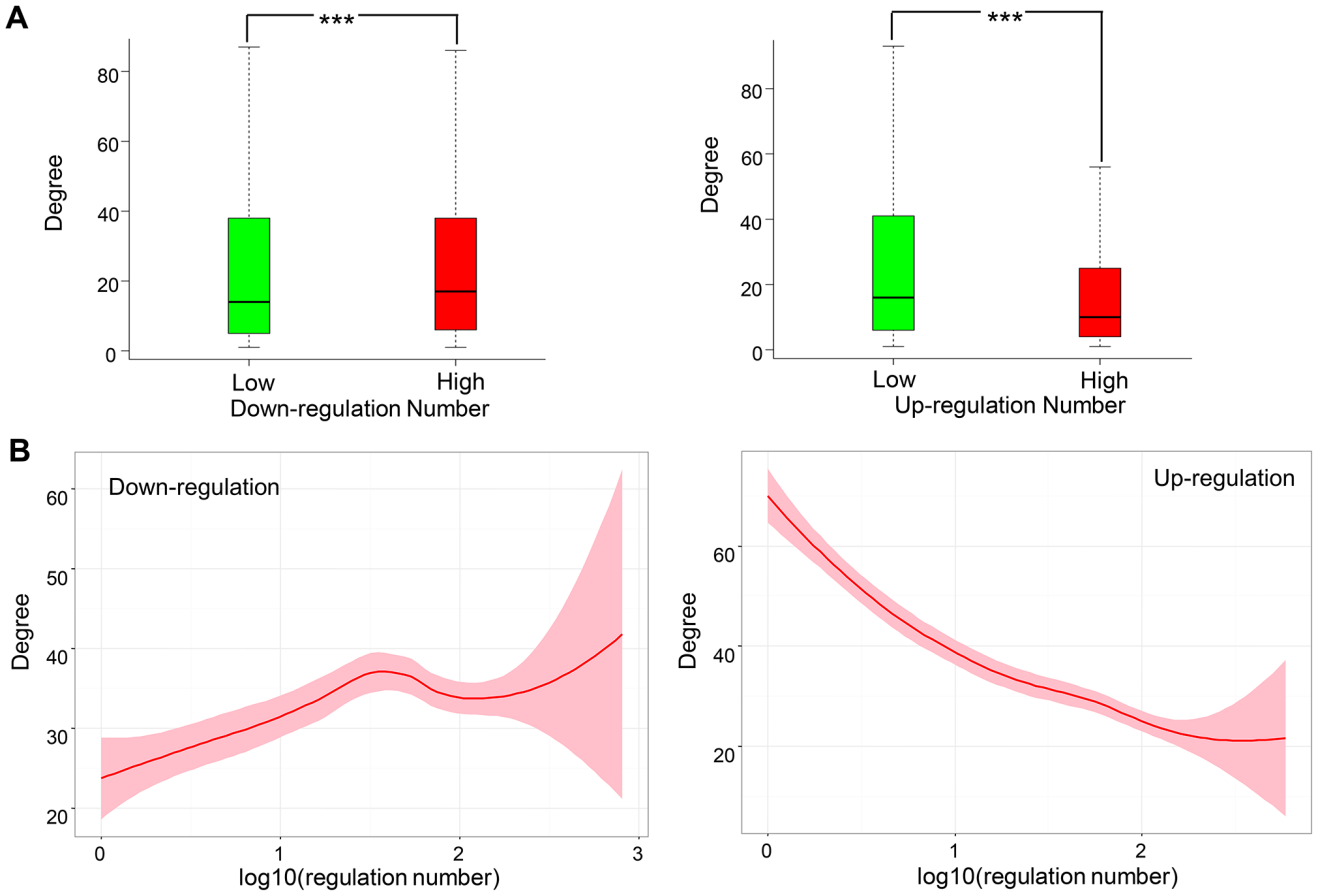
Interaction network degree analysis. (A) Comparison of degree in PPI network of down_l versus down_h (left) and up_l versus up_h (right). (B) The correlation between degree and up/down-regulation number. The correlation curve is plotted by using the LOESS smoothing techniques and the shade indicates the confidence interval.

The topology property of a specific gene in a PPI network could reflect its importance in some biological processes. Here, we downloaded the human PPI network from BioGrid and calculated degree (number of interaction partners) of every gene for up_h versus up_l and down_h versus down_l comparisons. As a result, the degree of down_l was found significantly lower than that of down_h (Fig 6A left, *P-Value* = 0.005901 by Wilcoxon test), whereas, the opposite tendency was observed when comparing up_l and up_h (Fig 6A right, *P-Value* = 3.929e-31, Wilcoxon test). We confirmed these observations by correlation analysis (Fig 6B). Significant positive correlation was obtained between down-regulation number and the corresponding degree in PPI network (Spearman correlation coefficient = 0.1174, *P-Value* = 2.997e-32), but significant negative correlation was obtained between up-regulation number and the degree (Spearman correlation coefficient = -0.2468, *P-Value* = 1.186e-139). This result is consistent with the study of Kotlyar *et al* [31], which indicat that down-regulated genes after drug treatment tend to have higher degree than those of up-regulated genes.

We next explored the subcellular localization differences between genes with different DEN. As a result, T1000D have higher gene proportion in extracellular region and membrane, and lower gene proportion in cytoplasm and nucleus than L1000D (S4 Fig; *P-Value* = 1.681e-43 by Chi-squared test). While the opposite results, i.e. higher gene proportion in cytoplasm and nucleus and lower gene proportion in extracellular region and membrane in T1000U than that of L1000U were obtained (S4 Fig; *P-Value* = 2.972e-12 by Chi-squared test). This result indicates that the drugs tend to frequently activate the expression of inner cell proteins but repress the expression of secreted proteins. However, as the transcriptome data are obtained from cancer cell line, whether such observation is cancer-specific requires further validation.

### Plausible implication and limitation of our analysis for drug discovery

Good drugs are usually effecting on specific gene or pathway [1], whereas, one gene would be regulated by multi drugs. Successful prediction of drug target genes have been achieved by using network or machine learning methods [15, 32, 33]. However, large-scale transcriptome survey like CMAP project has demonstrated that one drug could regulate the expression of many genes [18]. Indeed, the crosstalk between drug targets could have implication in drug synergistic combination effect or novel drug-target interactions [16, 34]. Therefore, systemic comparative analysis of genes regulated by multiple drugs would be useful for the development of new drugs. On the one hand, these promiscuous drug responsive genes can act as the secondary drug targets to amplify the drug effect. This effect is of particular interest for cancer therapy as drug toxicity issue is more tolerated in such cases. Indeed, our functional enrichment analysis showed that both up_h and down_h could be associated with transcriptional or translational functions, implying these genes may assist the propagation of drug signal through the gene regulation cascades. However, on the other hand, unwanted perturbation of gene expression could induce adverse side effect. Our analysis indicated the higher dSNP proportion for both up_h and down_h genes. When drug invokes differential expression of these genes with pattern similar to that under disease condition, adverse side-effect becomes more likely. Therefore, it is clear that the next step is to design a method to distinguish these two opposite effects of the high DEN genes. Although such topic is beyond the scope of this study, our analysis provided helpful indication about it. We have found the positive correlation between up-regulation number and tissue expression specificity, and negative correlation between down-regulation number and protein interaction network degree and phyletic age. Therefore, the unwanted activation of house-keeping genes or highly conserved genes involved in basic cellular processes could be considered as the indicator of adverse effect.

There are also obvious limitations of our analysis. First, the CMAP transcriptome data used were all from cancer cell lines, which could result in bias toward cancer-specific observations. So further studies are still needed for the systemic analysis of other types of diseases. Moreover, our study takes the effects of all drugs together rather than separately, it would be useful for the exploration of effects of specific drug if its influenced genes are analyzed individually. Finally, only the differential expression of coding genes was considered in our analysis. However, many non-coding RNAs like microRNAs have been shown to be associated with diseases [35], and the prediction of diseased-associated microRNAs could provide novel knowledge for therapeutic targets [36, 37]. It is therefore interesting to analyze the frequently regulated miRNAs by drugs in the future, in order to explore the regulation of drug response mediated by the non-coding RNAs.

## Conclusion

In this study, we conducted comparative analysis of genes with different DENs for their baseline expression, evolution, functions, topology properties and disease SNP density. We summarized the differences between genes more or less likely affected by drugs, as well as the differences between up- and down-regulated genes after drug treatment. Briefly, genes frequently regulated by drugs are more likely to be associated with disease-related functions and mutations, but the extensively up-regulated genes by drugs are not likely to be the house-keeping genes with prominently high conservation, high interaction network degree and wide tissue expression pattern. With the accumulation of other types of omics data, more comprehensive analysis of the drug effect by combing transcriptome data and other omics data will become feasible in the future and it should be promising for drug target discovery and side effect prediction.

## Supporting information

S1 Fig The sample illustration of genes with high and low DENs. (A) The heatmap illustrating the logarithmic transformed fold change (lnFC) across different drug treatment conditions, with respect to the top 20 and the last 20 genes from the down-regulation number distribution. (B) The heatmap illustrating the lnFC across different drug treatment conditions, with respect to the top 20 and the last 20 genes from the up-regulation number distribution. (C) Venn diagram showing the overlap between the up_h and down_h genes.
(TIF)

S2 Fig Functional enrichment analysis of genes with different down-regulation numbers. The enriched function of top 1000 (T1000D) and last 1000 genes (L1000D) from the down-regulation number distribution were listed and compared. T1000D were mainly involved in functions related to cancer development, while L1000D were significantly enriched in the progression of nervous system diseases.
(TIF)

S3 Fig Functional enrichment analysis of genes with different up-regulation numbers. The enriched function of top 1000 (T1000U) and last 1000 genes (L1000U) from the up-regulation number distribution were listed and compared. T1000U were mainly involved in functions related to cancer development, while L1000U were significantly enriched in the progression of nervous system diseases.
(TIF)

S4 Fig Subcellular locations comparison. The percentages of genes with different subcellular localization were illustrated for T1000D and L1000D comparison (left) and T1000U versus L1000U comparison (right). The percentages on the graphics represented the fractions of T1000D/L1000D and T1000U/L1000U in the total of genes in each DEN group.
(TIF)
