# Supplementary figures and images for: Comparative analysis of genes frequently regulated by drugs based on connectivity map transcriptome data
Source: PLoS One. 2017 Jun 2;12(6):e0179037. doi: 10.1371/journal.pone.0179037 (PMC5456389; doi:10.1371/journal.pone.0179037)

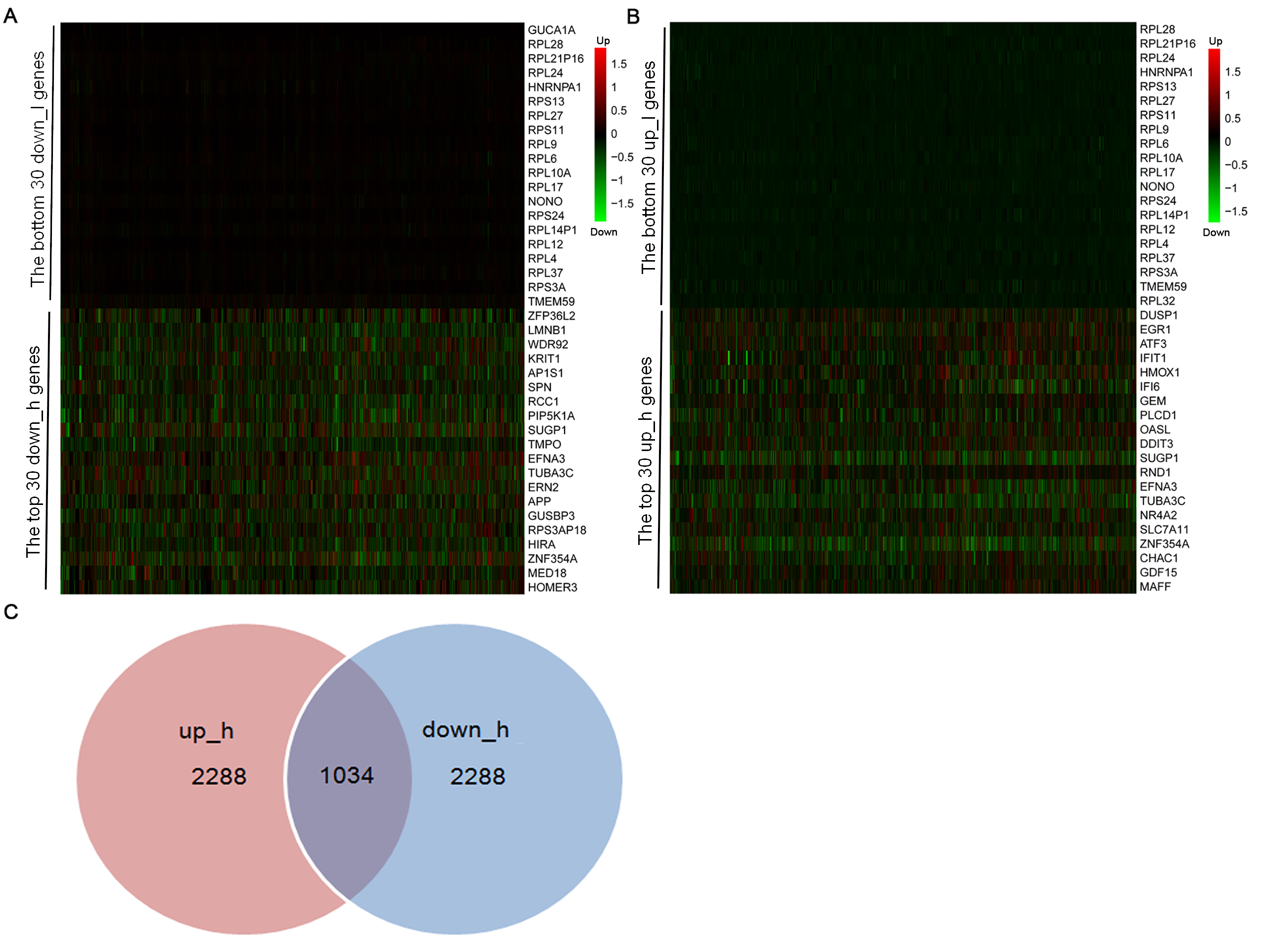

Supplement: S1 Fig — (A) The heatmap illustrating the logarithmic transformed fold change (lnFC) across different drug treatment conditions, with respect to the top 20 and the last 20 genes from the down-regulation number distribution. (B) The heatmap illustrating the lnFC across different drug treatment conditions, with respect to the top 20 and the last 20 genes from the up-regulation number distribution. (C) Venn diagram showing the overlap between the up_h and down_h genes. (TIF) [file pone.0179037.s001.tif]

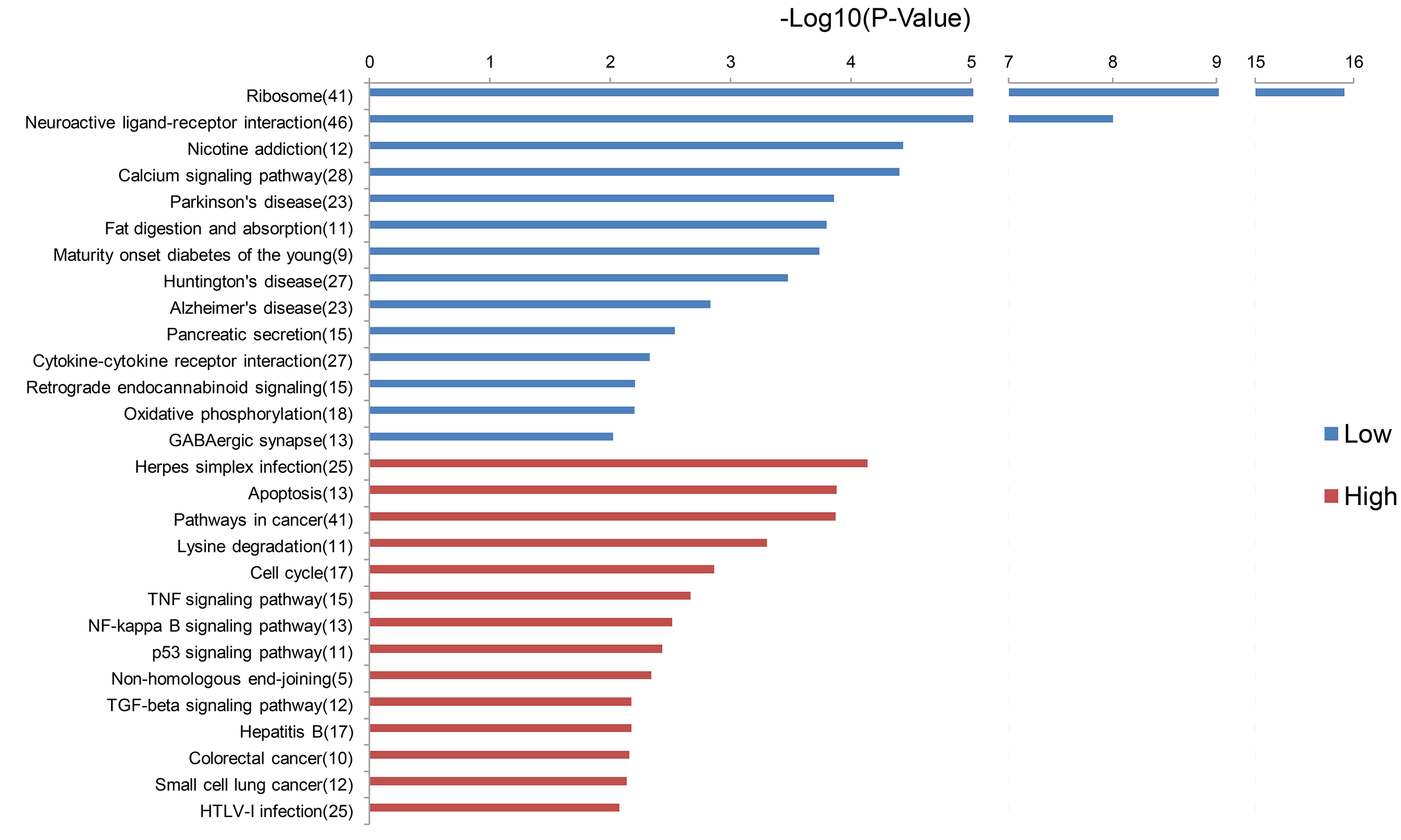

Supplement: S2 Fig — The enriched function of top 1000 (T1000D) and last 1000 genes (L1000D) from the down-regulation number distribution were listed and compared. T1000D were mainly involved in functions related to cancer development, while L1000D were significantly enriched in the progression of nervous system diseases. (TIF) [file pone.0179037.s002.tif]

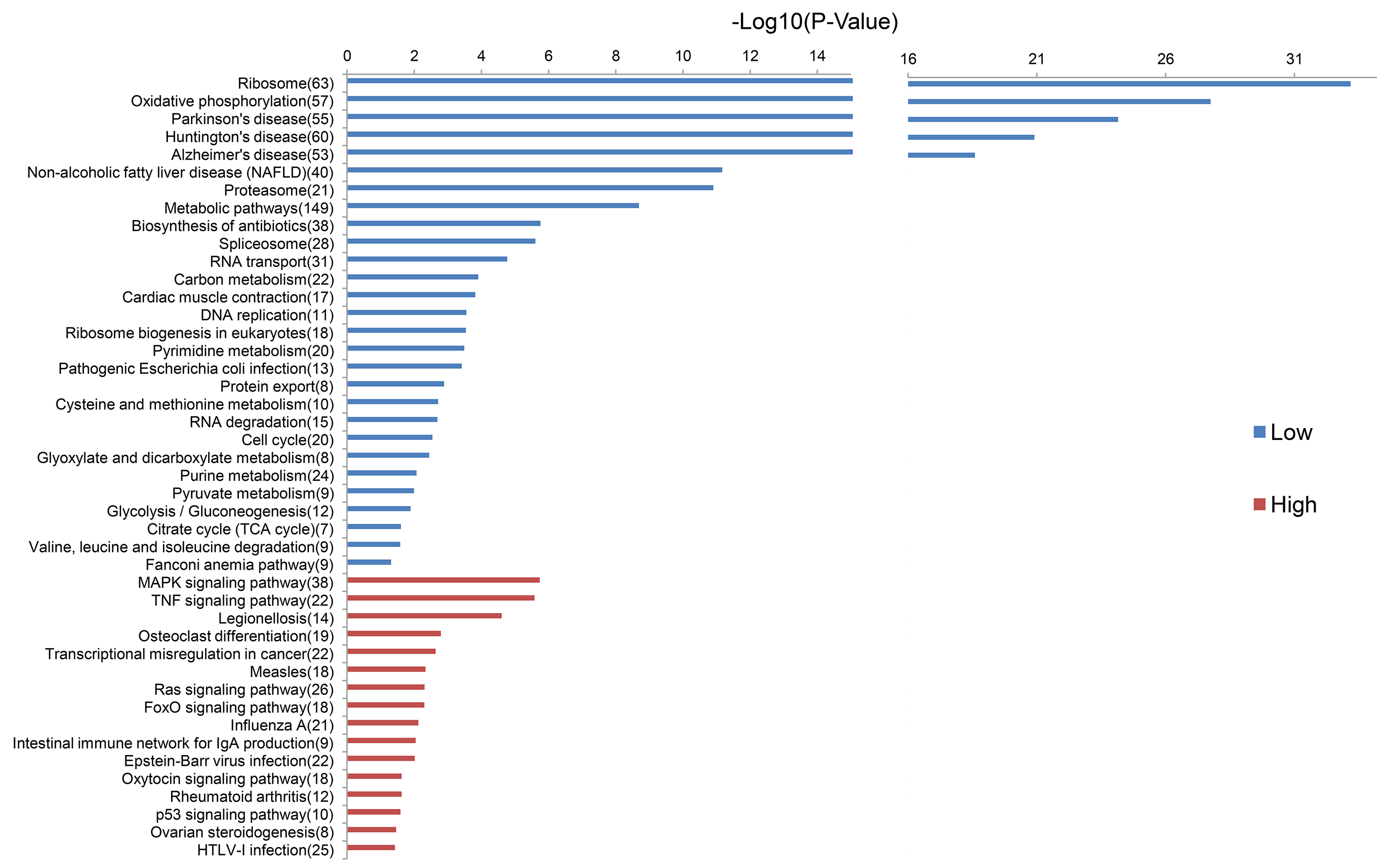

Supplement: S3 Fig — The enriched function of top 1000 (T1000U) and last 1000 genes (L1000U) from the up-regulation number distribution were listed and compared. T1000U were mainly involved in functions related to cancer development, while L1000U were significantly enriched in the progression of nervous system diseases. (TIF) [file pone.0179037.s003.tif]

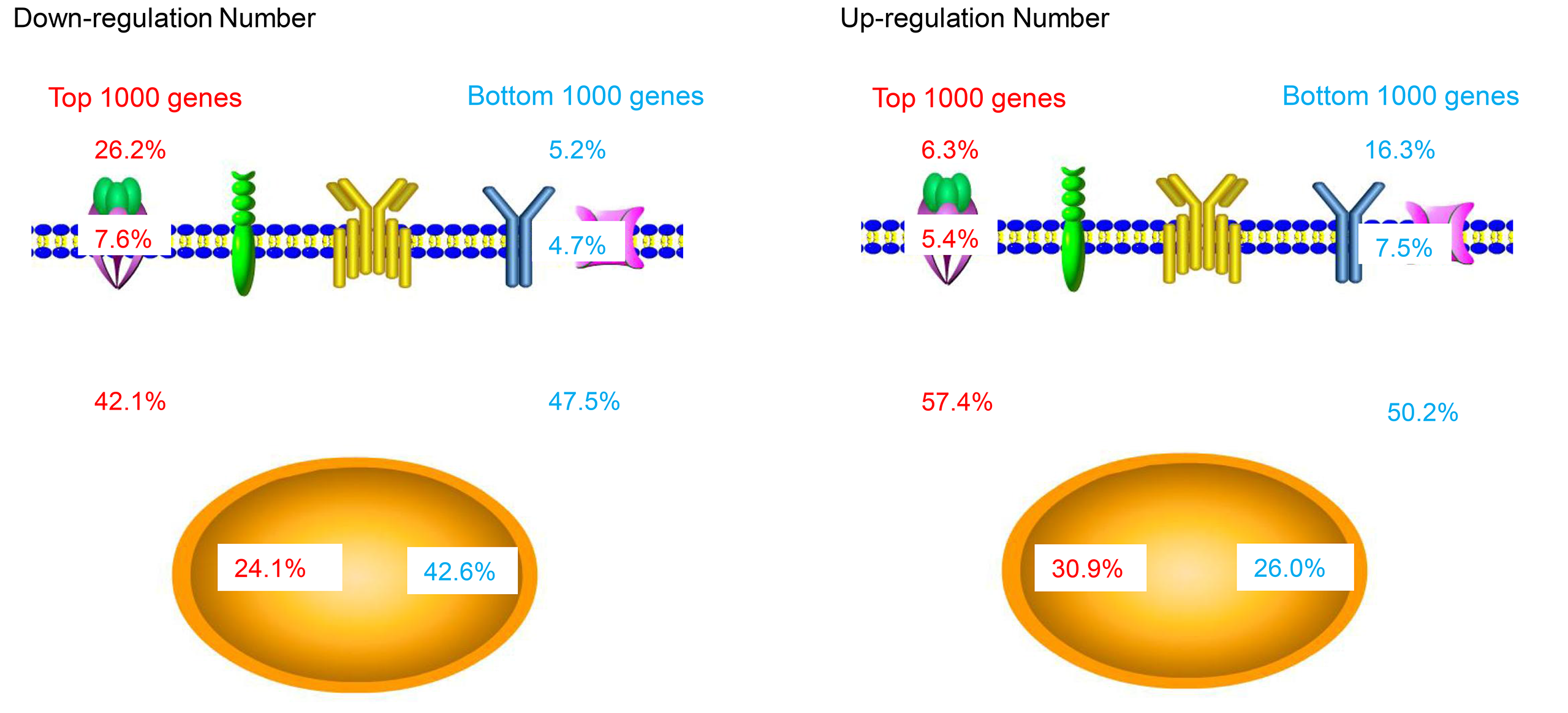

Supplement: S4 Fig — The percentages of genes with different subcellular localization were illustrated for T1000D and L1000D comparison (left) and T1000U versus L1000U comparison (right). The percentages on the graphics represented the fractions of T1000D/L1000D and T1000U/L1000U in the total of genes in each DEN group. (TIF) [file pone.0179037.s004.tif]
